# Prevalence of C‐shaped canals and **three‐rooted** mandibular molars in the Iranian population by using cone‐beam computed tomography

**DOI:** 10.1002/cre2.787

**Published:** 2023-10-03

**Authors:** Mina Shekarian, Masih Majlesi, Maryam Zare Jahromi

**Affiliations:** ^1^ School of Dentistry, Dental Research Center, Dental Research Institute Isfahan University of Medical Sciences Isfahan Iran; ^2^ School of Dentistry, Isfahan (Khorasgan) Branch Islamic Azad University Isfahan Iran; ^3^ Department of Endodontics, School of Dentistry, Isfahan (Khorasgan) Branch Islamic Azad University Isfahan Iran

**Keywords:** canal configuration, cone‐beam computed tomography, C‐shaped canals, three‐rooted

## Abstract

**Objectives:**

This study aimed to evaluate the prevalence of C‐shaped canals and three‐rooted mandibular molars in the Iranian population using cone‐beam computed tomography (CBCT).

**Materials and Methods:**

This study evaluated mandibular teeth on 292 CBCT scans of patients referred to the Department of Radiology, Faculty of Dentistry, Islamic Azad University, Isfahan, Iran. All CBCT scans were obtained with the same three‐dimensional CBCT scanner, and sections were reconstructed in all three planes (sagittal, axial, and coronal) with 1 mm slice thickness. A total of 291,402, and 200 first, second, and third molars, respectively, were evaluated. Data were analyzed using SPSS software (version 26.0).

**Results:**

Of 291 mandibular first molars, 0.7% were single‐rooted, 96.6% were two‐rooted, and 2.7% were three‐rooted. The prevalence of C‐shaped canals was 1.7% in mandibular first molars. Of 402 mandibular second molars, 8.5% were single‐rooted, 90.5% were two‐rooted, and 1% were three‐rooted. The prevalence of C‐shaped canals was 2% in mandibular second molars. Of 200 mandibular third molars, 21.5% were single‐rooted, 77.5% were two‐rooted, and 1% were three‐rooted. The prevalence of C‐shaped canals was 2% in mandibular third molars.

**Conclusion:**

In the present study, the majority of mandibular molars were two‐rooted, and three‐rooted mandibular molars were less common. The third and second mandibular molars both had an increased prevalence of C‐shaped canals. Mandibular first molars had the highest prevalence of three‐rooted molars.

## 1 INTRODUCTION

Knowledge about the normal root canal anatomy and its variations is imperative for successful root canal therapy (Torabinejad et al. 2023). Root canal anatomical complexities such as C‐shaped canals, complex anatomy of the pulp chamber, and the presence of additional root canals can complicate root canal therapy and pose a challenge for clinicians. Studies on anatomical complexities of the root canal system have used different methods for this purpose, such as tooth sectioning, microscopic evaluations, clearing techniques, periapical radiography, and cone‐beam computed tomography (CBCT). CBCT is reportedly the most accurate noninvasive technique for identification of C‐shaped canals (Baghbani et al., 2021; Patel et al., 2009; Shirkavand et al., 2016; Zhang et al., 2011; von Zuben et al., 2017).

The presence of two roots is the most common variant in mandibular molars. However, anatomical variations are frequently seen in mandibular molars, which can complicate the root canal treatment. Therefore, knowledge in this respect is imperative for dental clinicians.

Three‐rooted molars were first identified by Carabelli (1844). Radix entomolaris (RE) is the term used for an additional root located lingually, and radix paramolaris is the term used for an additional root located buccally. A high frequency (approximately 21%) of three‐rooted mandibular first molars has been detected in the Taiwanese (Chinese) population (Tu et al., 2007). Khurayzi et al. reported the prevalence of RE in permanent mandibular first molars to range from 2% to 6.07% in the Saudi Arabian population (Khurayzi et al., 2021). Another study reported a higher prevalence of RE in the Iranian population compared with Caucasian and European populations (Kuzekanani et al., 2017). The prevalence of three‐rooted mandibular molars in the Iranian population is estimated at 3% (Rahimi et al., 2017).

C‐shaped canals, which are defined as root canals shaped like the English letter C or a ribbon in transverse sections (Gulabivala et al., 2002; Weine, 1998), are also detected in mandibular molars, and are of great concern in endodontic treatment. Poor knowledge about the C‐shaped root canal anatomy can lead to missing a canal, root canal perforation, or inadequate debridement of the root canals (Nair, 2006). Dental clinicians may face several challenges in endodontic treatment of C‐shaped canals, such as the difficult negotiation of the root canal orifice, the need for additional irrigation protocols or specific obturation techniques, and the necessity of using an electronic apex locator (Jafarzadeh et al., 2017; Jafarzadeh & Wu, 2007). Although some studies emphasize that C‐shaped canals are not highly common (Madani et al., 2017; Naseri et al., 2013; Rahimi et al., 2017, 2008), they are not rare to find either (Nair, 2006; Rahimi et al., 2017).

Despite the availability of several studies regarding the prevalence of C‐shaped canals in the Iranian population, the reported results have been conflicting (Akhlaghi et al., 2016; Haddadi et al., 2019; Janani et al., 2018; Madani et al., 2017; Rad et al., 2020; Sarraf et al., 2022; Seo et al., 2012; Tafakhori et al., 2022). Inconsistencies in the reported results are most likely caused by variations in statistical populations and study methodologies. For example, a wide range of variation exists in the reported prevalence rates for C‐shaped canals in second molars (Haddadi et al., 2019; Janani et al., 2018; Madani et al., 2017; Ostevar Rad et al., 2020). Also, previous studies on this topic mostly had a small sample size (Akhlaghi et al., 2016; Janani et al., 2018; Madani et al., 2017; Sarraf et al., 2022; Tafakhori et al., 2022). Therefore, further studies with a larger sample size are required in this respect to address the inconsistencies in the published studies, and find more accurate results, because a larger sample size would minimize the risk of errors. Additionally, unlike some previous research (Nie et al., 2013; Rahimi et al., 2008; Shirkavand et al., 2016), high‐resolution CBCT was used in this study because it is known as the most accurate technique for identifying C‐shaped canals (Baghbani et al., 2021).

This study used archival data from the Department of Radiology, Faculty of Dentistry, Islamic Azad University, Isfahan, Iran to assess the prevalence of two main anatomical variations of mandibular molars found on CBCT scans namely C‐shaped canals and three‐rooted mandibular molars. The results of the present study can enhance the knowledge of dentists about the prevalence of these two variations in the target population and increase the success of endodontic treatment.

## 2 MATERIALS AND METHODS

This descriptive cross‐sectional study evaluated 292 CBCT scans of patients (141 males and 151 females) referred to the Department of Radiology, Faculty of Dentistry, Islamic Azad University, Isfahan, Iran between 2013 and 2021 that were selected by simple random sampling. The total sample size was calculated assuming *α* .05 = and study power of 90%.

The CBCT scans had been requested as part of the dental treatment of patients (not related to this study). The study protocol was approved by the ethics committee of the Islamic Azad University of Isfahan (code: ir.iau.khuisf.rec.1401.174). For data collection, a form was designed with two parts. The first part included the demographic information of patients, and the second part included the number and side of each mandibular molar, the number of roots, root canal configuration, and the date of taking the CBCT scan.

A total of 291 first molars, 402 s molars, and 200 third molars were selected based on the following inclusion criteria: Iranian patients with fully erupted molars, and mature apices. Teeth with root resorption, incompletely formed roots, calcification, fracture, major artifacts, amalgam or metal restorations, crowns, or previous endodontic treatment were excluded. The teeth were evaluated by three examiners: a dentistry graduated student, an endodontist, and an oral and maxillofacial radiologist. The three examiners were calibrated for the detection of C‐shaped canals and three‐rooted mandibular molars by evaluating the cases in all three planes together. The three examiners independently assessed the CBCT scans. In case of disagreement and inability to reach a consensus, the oral and maxillofacial radiologist would make the final decision.

All images were obtained using the same three‐dimensional (3D) CBCT scanner (D‐64625; Sirona Dental Systems GmbH), with the exposure settings of 85 kV voltage, 5.2 mA amperage, and 0.180 mm voxel size.

All images were observed on a 22‐inch flat‐panel monitor (LG 22MP57HQ; LG Electronics), with a screen refresh rate of 60 Hz, and true color (32 bit).

OnDemand 3D software was used to evaluate the CBCT images. Each tooth was evaluated in all three planes (sagittal, axial, and coronal) to identify the C‐shaped canals and number of roots. The slice thickness was 1 mm for all sections in all three planes. The final decision regarding the presence/absence of C‐shaped canals and three‐rooted teeth was made based on the consensus of the three examiners.

**Figure 1:**
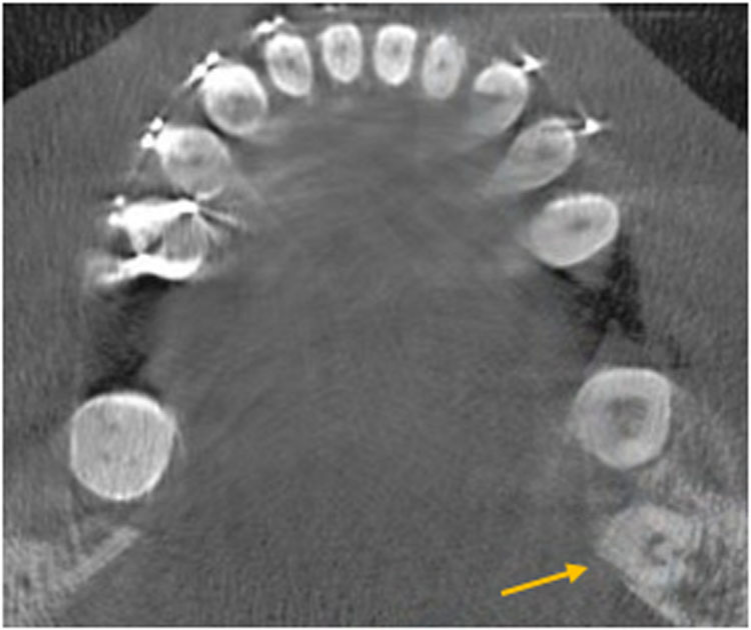
Axial CBCT view of a representative mandibular third molar with a C‐shaped canal. CBCT, cone‐beam computed tomography.

The examiners were allowed to adjust the contrast and magnification of images using the software enhancement tools. In the software environment, the teeth were aligned in an upright position, and the axial level was adjusted at the level of the root canal orifice. Each tooth diagnosed with a C‐shaped canal was precisely re‐evaluated corono‐apically on axial sections from the cementoenamel junction to the radiographic root apex to ensure the presence of a C‐shaped canal and determine its class according to the classification system proposed by Fan et al. (2004). Figure 1 shows a representative image of the mandibular third molar with a C‐shaped canal. In addition, the sagittal and coronal planes were evaluated several times to assess the presence/absence of RE. The number of roots, side of the mandible, and root canal anatomy were recorded in the respective form.

To assess the inter‐ and intraexaminer agreements, the three examiners re‐evaluated 25% of the images randomly 3 weeks after the first evaluation.

The number of roots and C‐shaped canals and their correlation with gender were analyzed by the *χ* ^2^ test using SPSS software (version 26.0). The level of statistical significance was set at .05.

## 3 RESULTS

A total of 1248 CBCT images were initially evaluated; out of which, 292 CBCT scans were selected based on the eligibility criteria. A total of 893 teeth were evaluated on the abovementioned 292 CBCT scans, including 291 mandibular first molars, 402 mandibular second molars, and 200 mandibular third molars. The Cohen's kappa coefficient ranged from 0.918 to 0.923 for intraexaminer agreement, and 0.934–0.952 for interexaminer agreement.

The prevalence of C‐shaped canal configuration and three‐rooted mandibular molars based on gender is presented in Table 1. Statistically, among mandibular first molars, two‐rooted teeth were more prevalent than other types (*p* < .001), and no significant difference was found between males and females in this respect (*p* > .05). Moreover, the prevalence of different root types did not differ significantly between males and females (*p* > .05).

**Table 1:** Prevalence of C‐shaped canals and root number in mandibular molars by gender.

	First molars (*N* = 291)				Second molars (*N* = 402)				Third molars (*N* = 200)			
	Single rooted	Two rooted	Three rooted	C‐shaped canal	Single rooted	Two rooted	Three rooted	C‐shaped canal	Single rooted	Two rooted	Three rooted	C‐shaped canal
Males	1 (0.7%)	141 (96.6%)	4 (2.7%)	0	15 (7.4%)	186 (91.6%)	2 (1%)	2 (1%)	15 (14.7%)	37 (85.3%)	0	2 (2%)
Females	1 (2.8%)	140 (96.6%)	4 (0.7%)	5 (3.4%)	19 (9.5%)	178 (89.4%)	2 (1%)	6 (3%)	28 (28.6%)	68 (69.4%)	2 (2%)	2 (2%)
Total	2 (0.7%)	281 (96.6%)	8 (2.7%)	5 (1.7%)	34 (8.5%)	364 (90.5%)	4 (1%)	8 (2%)	43 (21.5%)	155 (77.5%)	2 (1%)	4 (2%)
*p*‐Value	1.00	1.00	1.00	.06	.73	.73	.73	.28	.01*	.01*	.01*	1.00

Assessment of CBCT images revealed that two‐rooted teeth were more common among the 402 mandibular second molars (*p* < .001), and their prevalence was significantly higher in males than females (*p* < .001).

Among the 200 mandibular third molars evaluated in this study, the prevalence of two‐rooted teeth was significantly higher than other types (*p* < .001).

The number of single‐rooted teeth in mandibular second and third molars was significantly higher in females than males (*p* < .05).

C‐shaped canals were equally more common in mandibular second molars and third molars (2%). However, three‐rooted molars were the most common in mandibular first molars (2.7%).

Although the prevalence of C‐shaped canal configuration was slightly higher in females than males, there was no significant correlation between gender and frequency of C‐shaped canal configuration (*p* > .05).

In addition, the *χ* ^2^ test demonstrated an insignificant correlation between the number of roots in mandibular third molars and gender (*p* = .10). As shown in Table 1, although the prevalence of single‐rooted teeth in females was approximately twice the rate in males (*p* = .665), the prevalence of two‐rooted teeth was higher in males than females (*p* = .564).

## 4 DISCUSSION

Assessment of root canal system configuration is always a challenge for dental clinicians. In addition, knowledge about the precise root canal anatomy is imperative to maximize the quality of treatment. Since racial variations affect the root canal configuration, and considering the complex root canal anatomy of mandibular molars, this study aimed to assess the prevalence of C‐shaped canals and three‐rooted mandibular molars in the Iranian population, especially because not many studies have investigated the anatomy of mandibular third molars.

CBCT, peripheral quantitative CT, spiral CT, plain digital radiography, contrast medium‐enhanced digital radiography, canal staining, and tooth‐clearing techniques are among the suggested modalities by Neelakantan et al. (2010) for the evaluation of canal types. Evidence shows that although the clearing technique is the gold standard for assessment of the root canal system, CBCT is as accurate as the tooth‐clearing technique for this purpose (Neelakantan et al., 2010). Also, it is noteworthy that CBCT is a noninvasive technique compared with other methods (Yang et al., 2013). In addition, since CBCT enables 3D assessment of the objects, its accuracy is higher than that of other modalities such as intraoral radiography. The accuracy of preapical radiography for evaluation of root canal configurations reportedly ranges from 36.6% to 40% while the accuracy of CBCT is estimated to be 76.6%–83.3% (Pinsky et al., 2006).

Many studies reported a significantly high prevalence of C‐shaped canals in mandibular second molars (Haddadi et al., 2019; Madani et al., 2017; Martins et al., 2019; Roy et al., 2019; Yang et al., 2021; Zheng et al., 2011; von Zuben et al., 2017). According to previous studies, the C‐shaped canal configuration is observed more frequently in Asian populations (Alfawaz et al., 2019; Duman et al., 2019; Hajihassani et al., 2017; Nie et al., 2013; Pan et al., 2019; Singh et al., 2022; Sirawut et al., 2021; Suzuki et al., 2015). The frequency of C‐shaped canals in mandibular second molars was reported to be more prevalent in the Chinese population than in other races (Fernandes et al., 2014; Roy et al., 2019; von Zuben et al., 2017). A literature review showed that the Lebanese population had a higher incidence of C‐shaped canals in mandibular second molars in comparison with other West‐Asian populations, including the Iranian population (Fernandes et al., 2014).

No consensus has reached on the relationship of gender and C‐shaped canal configuration. Of the studies conducted in Iran, some confirmed this correlation (Haddadi et al., 2019; Madani et al., 2017; Sarraf et al., 2022). Some studies indicated a higher frequency of C‐shaped canals in females (Haddadi et al., 2019; Madani et al., 2017); while some others reported a higher frequency of C‐shaped canals in males (Sarraf et al., 2022). In contrast, some other studies did not find a significant difference in the distribution of C‐shaped canals between males and females (Janani et al., 2018; Ostevar Rad et al., 2020). The present results were in agreement with the findings of those that did not find a significant correlation between gender and C‐shaped canal configuration (Janani et al., 2018; Ostevar Rad et al., 2020).

Most studies conducted in Asia did not find any significant correlation between three‐rooted molars and gender (Çolak et al., 2012; Gupta et al., 2017; Mathew & Soni, 2019; Nagaveni et al., 2018; Tafakhori et al., 2022; Tu et al., 2009, 2007). However, Riyahi et al. reported significant differences in the prevalence of additional roots in molars between males and females (Riyahi et al., 2019). They indicated that the prevalence of this variant was higher in females than males for the first molars, and higher in males than females for the second molars (Riyahi et al., 2019).

### 4.1 Mandibular first molars

The frequency of three roots is the highest among mandibular molars. Madani et al. (2017) and Rahimi et al. (2017) evaluated the presence of additional roots in mandibular first molars and found that 1.9% and 3% of mandibular first molars had three roots, respectively. However, this rate was 2.7% in the present study, which was closer to the value reported by Rahimi et al. (2017). Based on the present results, the frequency of C‐shaped root canals in the Iranian population was 1.7%, which was close to the rate reported by Madani et al. (2017) (1.2%).

### 4.2 Mandibular second molars

Although in most studies, C‐shaped root canal configuration was the most common anatomical variation found in mandibular second molars (Alfawaz et al., 2019; Naseri et al., 2013; Nie et al., 2013; Sarraf et al., 2022; Zhang et al., 2011), the present results indicated that the prevalence of C‐shaped canals in mandibular second molars was equal to that in mandibular third molars. The reported prevalence of C‐shaped canal configuration is widely variable, which might be attributed to different methods used for the identification of C‐shaped canals as well as the variations in sample size and study populations.

The prevalence of C‐shaped canals was reported to be 17.6%, 11.5%, and 21.4% on CBCT scans in studies by Madani et al. (2017), Haddadi et al. (2019), and Janani et al. (2018), respectively. However, in a study by Rahimi et al. (2008), who used extracted teeth, the prevalence of C‐shaped canals was reported to be 7.2%. The prevalence of C‐shaped canals was reported to be 6.96% in an ex vivo study by Akhlaghi et al. (2016), and also a systematic review (Naseri et al., 2013). This value was 2% in the present study which was close to the rate reported by Jahromi et al. (2013), (3%) using the clearing technique, probably because both studies were conducted on the Iranian population.

### 4.3 Mandibular third molars

To the best of the authors' knowledge, only one study published in 2017 investigated the morphology of mandibular third molars in the Iranian population (Kuzekanani et al., 2017).

It is noteworthy that in cases where first and second molars are absent, third molars may be used as an alternative to serve as an abutment for a fixed partial denture. In addition, third molars may participate in occlusion and enhance mastication when their opposing teeth are present in the dental arch. Therefore, the quality of root canal treatment of such teeth is highly important, especially because of difficult access and complicated root canal anatomy and morphology.

Kuzekanani et al. (2017) reported the incidence of C‐shaped canals to be 3.5%, which was higher than the rate in the present study (2%). Moreover, in their study, the percentage of three‐rooted molars was 5.5%; while this rate was 1% in the present study (Kuzekanani et al., 2017).

Ostevar Rad et al. (2020) had the largest sample size among the previous studies conducted in this regard in Iran. The sample size of the present study was larger than that of Ostevar Rad et al. (2020) by over 1.5 times. Moreover, to the best of the authors' knowledge, this study is the first to assess the prevalence of C‐shaped canals and three‐rooted molars by assessing mandibular first, second, and third molars altogether with a large sample size.

Considering the variations in the reported results in the literature, further studies are required to assess the prevalence of C‐shaped canals in mandibular molars in the Iranian population. Also, concerning the role of ethnicity in anatomical variations, regional studies are required on different ethnic groups residing in Iran, as well as nationwide studies with a large sample size on participants from different ethnic groups and geographical locations of Iran.

The distinction between C‐shaped canal types and subtypes, as well as the radix‐entomolaris and paramolaris, were not investigated in this study. Because they were not the main objectives of this study.

## 5 CONCLUSION

In the present study, the majority of mandibular molars were two‐rooted, and three‐rooted mandibular molars were less common. The third and second mandibular molars both had an increased prevalence of C‐shaped canals. Mandibular first molars had the highest prevalence of three‐rooted molars. This study did not find any correlation between gender and C‐shaped canal configuration or three‐rooted molars.

## 6 LIMITATIONS

This study was only conducted in one center in one city of Iran. Although multicenter studies on participants from different ethnic groups and geographical locations increase the generalizability of the results, it was not possible because of the difficulty to access to different centers. In addition, another issue of data gathering from multicenter was the impossibility of comparing data due to the use of different CBCT radiographic machines.

C‐shaped canal types and subtypes, as well as the distinction between radix‐entomolaris and paramolaris, were not investigated in this study because they were not the main objectives of this study.

## AUTHOR CONTRIBUTIONS

All authors substantially contributed to the conception and design of the study, acquisition of data, analysis, and interpretation of data, drafting of the article or revising it, and final approval of the version to be submitted. The detailed list of contributions is provided in this paper.

## CONFLICT OF INTEREST STATEMENT

The authors declare no conflict of interest.

## Data Availability

If anyone needs access to our data, please email the first author (shekarianmina@gmail.com).

## References

[cre2787-bib-0001] Akhlaghi, N. , Abbas, F. , Mohammadi, M. , Shamloo, M. K. , Radmehr, O. , Kaviani, R. , & Rakhshan, V. (2016). Radicular anatomy of permanent mandibular second molars in an Iranian population: A preliminary study. Dental Research Journal, 13(4), 362–366. 27605995 10.4103/1735-3327.187883 PMC4993065

[cre2787-bib-0002] Alfawaz, H. , Alqedairi, A. , Alkhayyal, A. K. , Almobarak, A. A. , Alhusain, M. F. , & Martins, J. N. R. (2019). Prevalence of C‐shaped canal system in mandibular first and second molars in a Saudi population assessed via cone beam computed tomography: A retrospective study. Clinical Oral Investigations, 23(1), 107–112. 10.1007/s00784-018-2415-0 29536188

[cre2787-bib-0003] Baghbani, A. , Bagherpour, A. , Ahmadis, Z. , Dehban, A. , Shahmohammadi, R. , & Jafarzadeh, H. (2021). The efficacy of five different techniques in identifying C‐shaped canals in mandibular molars. Australian Endodontic Journal, 47(2), 170–177. 10.1111/aej.12445 33030295

[cre2787-bib-0004] Carabelli, G. (1844). Systematic handbook of dentistry. Braumuller Seidel Publications.

[cre2787-bib-0005] Çolak, H. , Özcan, E. , & Hamidi, M. (2012). Prevalence of three‐rooted mandibular permanent first molars among the Turkish population. Nigerian Journal of Clinical Practice, 15(3), 306. 22960966 10.4103/1119-3077.100627

[cre2787-bib-0006] Duman, B. S. , Duman, S. , Bayrakdar, I. S. , Yasa, Y. , & Gumussoy, I. (2019). Cone‐beam computed tomography evaluation of C‐shape canals and longitudinal grooves of mandibular first and second molar teeth. Annals of Medical Research, 26(12), 2853–2858.

[cre2787-bib-0007] Fan, B. , Cheung, G. , Fan, M. , Gutmann, J. , & Bian, Z. (2004). C‐shaped canal system in mandibular second molars: Part I—Anatomical features. Journal of Endodontics, 30(12), 899–903. 15564874 10.1097/01.don.0000136207.12204.e4

[cre2787-bib-0008] Fernandes, M. , De Ataide, I. , & Wagle, R. (2014). C‐shaped root canal configuration: A review of literature. Journal of Conservative Dentistry, 17(4), 312–319. 25125841 10.4103/0972-0707.136437 PMC4127687

[cre2787-bib-0009] Gulabivala, K. , Opasanon, A. , Ng, Y.‐L. , & Alavi, A. (2002). Root and canal morphology of Thai mandibular molars. International Endodontic Journal, 35(1), 56–62. 11853239 10.1046/j.1365-2591.2002.00452.x

[cre2787-bib-0010] Gupta, A. , Duhan, J. , & Wadhwa, J. (2017). Prevalence of three rooted permanent mandibular first molars in Haryana (North Indian) population. Contemporary Clinical Dentistry, 8(1), 38. 28566849 10.4103/ccd.ccd_699_16 PMC5426164

[cre2787-bib-0011] Haddadi, A. , Azizi, H. , Haghani far, S. , & Hoshyari, N. (2019). Morphology and prevalence of C‐shaped canal in mandibular second molars in a population in North of Iran. Journal of Mazandaran University of Medical Sciences, 29(178), 141–147.

[cre2787-bib-0012] Hajihassani, N. , Roohi, N. , Madadi, K. , Bakhshi, M. , & Tofangchiha, M. (2017). Evaluation of root canal morphology of mandibular first and second premolars using cone beam computed tomography in a defined group of dental patients in Iran. Scientifica, 2017, 1–7. 10.1155/2017/1504341 PMC5734008 29348968

[cre2787-bib-0013] Jafarzadeh, H. , Beyrami, M. , & Forghani, M. (2017). Evaluation of conventional radiography and an electronic apex locator in determining the working length in C‐shaped canals. Iranian Endodontic Journal, 12(1), 60. 28179926 10.22037/iej.2017.12 PMC5282381

[cre2787-bib-0014] Jafarzadeh, H. , & Wu, Y.‐N. (2007). The C‐shaped root canal configuration: A review. Journal of Endodontics, 33(5), 517–523. 17437864 10.1016/j.joen.2007.01.005

[cre2787-bib-0015] Jahromi, M. Z. , Golestan, F. J. , Esmaeil, M. M. , MoouaviZahed, S. , & Sarami, M. (2013). Root and canal morphology of mandibular second molar in an Iranian population by clearing method. Journal of Dentistry, 14(2), 78. 24724124 PMC3977544

[cre2787-bib-0016] Janani, M. , Rahimi, S. , Jafari, F. , Johari, M. , Nikniaz, S. , & Ghasemi, N. (2018). Anatomic features of C‐shaped mandibular second molars in a selected Iranian population using CBCT. Iranian Endodontic Journal, 13(1), 120–125. 29692847 10.22037/iej.v13i1.17286 PMC5800453

[cre2787-bib-0061] Khurayzi, T. A. , Beleges, E. M. , & Dallak, S. A. (2021). The prevalence ofradix entomolaris (RE) in the mandibular permanent first molars among the Saudiarabian population—a systematic review. Saudi Journal of Oral and Dental Research, 6(1), 22–30.

[cre2787-bib-0017] Kuzekanani, M. , Walsh, L. J. , Haghani, J. , & Kermani, A. Z. (2017). Radix entomolaris in the mandibular molar teeth of an Iranian population. International Journal of Dentistry, 2017, 1–4. 10.1155/2017/9364963 PMC5379090 28421115

[cre2787-bib-0018] Madani, Z. S. , Mehraban, N. , Moudi, E. , & Bijani, A. (2017). Root and canal morphology of mandibular molars in a selected Iranian population using cone‐beam computed tomography. Iranian Endodontic Journal, 12(2), 143. 28512476 10.22037/iej.2017.29 PMC5431731

[cre2787-bib-0019] Martins, J. N. R. , Marques, D. , Silva, E. J. N. L. , Caramês, J. , Mata, A. , & Versiani, M. A. (2019). Prevalence of C‐shaped canal morphology using cone beam computed tomography—A systematic review with meta‐analysis. International Endodonotic Journal, 52(11), 1556–1572. 10.1111/iej.13169 31215045

[cre2787-bib-0020] Mathew, M. , & Soni, A. (2019). Prevalence of three‐rooted primary mandibular first molars in Karnataka (South Indian) population. International Journal of Pedodontic Rehabilitation, 4(1), 6.

[cre2787-bib-0021] Nagaveni, N. , Poornima, P. , Valsan, A. , & Mathew, M. (2018). Prevalence of three‐rooted primary mandibular second molars in Karnataka (South Indian) population. International Journal of Pedodontic Rehabilitation, 3(1), 33.

[cre2787-bib-0022] Nair, P. N. R. (2006). On the causes of persistent apical periodontitis: A review. International Endodontic Journal, 39(4), 249–281. 16584489 10.1111/j.1365-2591.2006.01099.x

[cre2787-bib-0023] Naseri, M. , Haghighi, A. K. , Kharazifard, M. J. , & Khavid, A. (2013). Prevalence of C‐shaped root canals in Iranian population: A systematic review. Journal of Dentistry, 10(2), 186–196. 23724219 PMC3666080

[cre2787-bib-0024] Neelakantan, P. , Subbarao, C. , & Subbarao, C. V. (2010). Comparative evaluation of modified canal staining and clearing technique, cone‐beam computed tomography, peripheral quantitative computed tomography, spiral computed tomography, and plain and contrast medium–enhanced digital radiography in studying root canal morphology. Journal of Endodontics, 36(9), 1547–1551. 20728725 10.1016/j.joen.2010.05.008

[cre2787-bib-0025] Nie, Y. K. , Bakar, W. Z. W. , & Alam, M. K. (2013). The occurrence of C‐shaped root canal in Malaysian population. Bangladesh Journal of Medical Science, 12(3), 286–290.

[cre2787-bib-0027] Ostevar Rad, F. , Mousavi, E. , Musapoor, N. , Maleki, D. , & Khatibi, N. (2020). Prevalence of C‐shaped canals in anterior and posterior teeth of Iranian population using cone beam computed tomography. Avicenna Journal of Dental Research, 12(2), 58–62.

[cre2787-bib-0028] Pan, J. Y. Y. , Parolia, A. , Chuah, S. R. , Bhatia, S. , Mutalik, S. , & Pau, A. (2019). Root canal morphology of permanent teeth in a Malaysian subpopulation using cone‐beam computed tomography. BMC Oral Health, 19(1), 14. 30642318 10.1186/s12903-019-0710-z PMC6332542

[cre2787-bib-0029] Patel, S. , Dawood, A. , Whaites, E. , & Pitt Ford, T. (2009). New dimensions in endodontic imaging: Part 1. Conventional and alternative radiographic systems. International Endodontic Journal, 42(6), 447–462. 19298577 10.1111/j.1365-2591.2008.01530.x

[cre2787-bib-0030] Pinsky, H. , Dyda, S. , Pinsky, R. , Misch, K. , & Sarment, D. (2006). Accuracy of three‐dimensional measurements using cone‐beam CT. Dentomaxillofacial Radiology, 35(6), 410–416. 17082331 10.1259/dmfr/20987648

[cre2787-bib-0031] Rahimi, S. , Mokhtari, H. , Ranjkesh, B. , Johari, M. , Frough Reyhani, M. , Shahi, S. , & Seif Reyhani, S. (2017). Prevalence of extra roots in permanent mandibular first molars in Iranian population: A CBCT analysis. Iranian Endodontic Journal, 12(1), 70–73. 28179928 10.22037/iej.2017.14 PMC5282383

[cre2787-bib-0032] Rahimi, S. , Shahi, S. , Lotfi, M. , Zand, V. , Abdolrahimi, M. , & Es'haghi, R. (2008). Root canal configuration and the prevalence of C‐shaped canals in mandibular second molars in an Iranian population. Journal of Oral Science, 50(1), 9–13. 18403877 10.2334/josnusd.50.9

[cre2787-bib-0033] Riyahi, A. M. , Alssum, K. , Hadadi, H. , Alsayyari, A. , Alebrah, T. , & Aljarbou, F. (2019). Prevalence of three‐rooted mandibular permanent first and second molars in the Saudi population. The Saudi Dental Journal, 31(4), 492–495. 31695298 10.1016/j.sdentj.2019.04.010 PMC6823794

[cre2787-bib-0034] Roy, A. , Astekar, M. , Bansal, R. , Gurtu, A. , Kumar, M. , & Agarwal, L. (2019). Racial predilection of C‐shaped canal configuration in the mandibular second molar. Journal of Conservative Dentistry, 22(2), 133. 31142981 10.4103/JCD.JCD_369_18 PMC6519192

[cre2787-bib-0035] Sarraf, P. , Mohammadi, S. , Moghaddamzade, B. , & Khosraviani, F. (2022). Root canal anatomy and morphology evaluation of mandibular molars according to gender by cone‐beam computed tomography in Iranian population. International Journal of Applied Dental Sciences, 8(1), 307–312.

[cre2787-bib-0036] Seo, D. G. , Gu, Y. , Yi, Y. A. , Lee, S. J. , Jeong, J. S. , Lee, Y. , Chang, S. W. , Lee, J. K. , Park, W. , Kim, K. D. , & Kum, K. Y. (2012). A biometric study of C‐shaped root canal systems in mandibular second molars using cone‐beam computed tomography. International Endodontic Journal, 45(9), 807–814. 22432971 10.1111/j.1365-2591.2012.02037.x

[cre2787-bib-0037] Shirkavand, S. , Zare Jahromi, M. , Saei, M. R. , Ruzbeh, R. , & Moghaddam, R. (2016). Evaluation of root and canal morphology of mandibular first molars: A clearing method in an Iranian population. Avicenna Journal of Dental Research, 8(3), 3.

[cre2787-bib-0038] Singh, T. , Kumari, M. , Kochhar, R. , & Iqbal, S. (2022). Prevalence of C‐shaped canal and related variations in maxillary and mandibular second molars in the Indian subpopulation: A cone‐beam computed tomography analysis. Journal of Conservative Dentistry, 25(5), 531. 36506623 10.4103/jcd.jcd_234_22 PMC9733544

[cre2787-bib-0039] Sirawut, H.‐u. , Sunpatch, B. , Chidnarong, A. , Pornthip, T. , Pakjira, T. , & Apichai, O. (2021). Prevalence of C‐shaped canals and three‐rooted mandibular molars using CBCT in a selected Thai population. Iranian Endodontic Journal, 16, 97–102. 36704226 10.22037/iej.v16i2.30527 PMC9709890

[cre2787-bib-0040] Suzuki, M. , Tsujimoto, Y. , & Kondo, S. (2015). Morphological variations of the root canal system in C‐shaped roots of the mandibular second molar in a Japanese population. International Journal of Oral‐Medical Sciences, 13(3), 81–88.

[cre2787-bib-0041] Tafakhori, Z. , Rafiei, M. , & Paper, R. (2022). Frequency of radix molaris in mandibular first and second molars using cone‐beam computed tomography images in a selected Iranian population. Caspian Journal of Dental Research, 11(2), 124–129.

[cre2787-bib-0042] Torabinejad, M. , Fouad, A. , & Shabahang, S. (2023). Endodontics: Principles and practice. Elsevier.

[cre2787-bib-0043] Tu, M.‐G. , Huang, H.‐L. , Hsue, S.‐S. , Hsu, J.‐T. , Chen, S.‐Y. , Jou, M.‐J. , & Tsai, C. C. (2009). Detection of permanent three‐rooted mandibular first molars by cone‐beam computed tomography imaging in Taiwanese individuals. Journal of Endodontics, 35(4), 503–507. 19345794 10.1016/j.joen.2008.12.013

[cre2787-bib-0044] Tu, M.‐G. , Tsai, C.‐C. , Jou, M.‐J. , Chen, W.‐L. , Chang, Y.‐F. , Chen, S.‐Y. , & Cheng, H. W. (2007). Prevalence of three‐rooted mandibular first molars among Taiwanese individuals. Journal of Endodontics, 33(10), 1163–1166. 17889682 10.1016/j.joen.2007.07.020

[cre2787-bib-0045] Weine, F. S. (1998). The C‐shaped mandibular second molar: Incidence and other considerations. Journal of Endodontics, 24(5), 372–375. 9641116 10.1016/s0099-2399(98)80137-4

[cre2787-bib-0046] Yang, H. , Tian, C. , Li, G. , Yang, L. , Han, X. , & Wang, Y. (2013). A cone‐beam computed tomography study of the root canal morphology of mandibular first premolars and the location of root canal orifices and apical foramina in a Chinese subpopulation. Journal of Endodontics, 39(4), 435–438. 23522531 10.1016/j.joen.2012.11.003

[cre2787-bib-0047] Yang, S. E. , Lee, T. Y. , & Kim, K. J. (2021). Prevalence and morphology of C‐shaped canals: A CBCT analysis in a Korean population. Scanning, 2021, 1–8. 10.1155/2021/9152004 PMC8181116 34131465

[cre2787-bib-0048] Zhang, R. , Wang, H. , Tian, Y.‐Y. , Yu, X. , Hu, T. , & Dummer, P. M. H. (2011). Use of cone‐beam computed tomography to evaluate root and canal morphology of mandibular molars in Chinese individuals. International Endodontic Journal, 44(11), 990–999. 21658074 10.1111/j.1365-2591.2011.01904.x

[cre2787-bib-0049] Zheng, Q. , Zhang, L. , Zhou, X. , Wang, Q. , Wang, Y. , Tang, L. , Song, F. , & Huang, D. (2011). C‐shaped root canal system in mandibular second molars in a Chinese population evaluated by cone‐beam computed tomography. International Endodontic Journal, 44(9), 857–862. 21599707 10.1111/j.1365-2591.2011.01896.x

[cre2787-bib-0050] von Zuben, M. , Martins, J. N. R. , Berti, L. , Cassim, I. , Flynn, D. , Gonzalez, J. A. , Gu, Y. , Kottoor, J. , Monroe, A. , Rosas Aguilar, R. , Marques, M. S. , & Ginjeira, A. (2017). Worldwide prevalence of mandibular second molar C‐shaped morphologies evaluated by cone‐beam computed tomography. Journal of Endodontics, 43(9), 1442–1447. 28734652 10.1016/j.joen.2017.04.016

